# Squamous Cell Carcinoma With Sarcomatous Stroma of the Mesopharynx

**DOI:** 10.1155/DTE.5.125

**Published:** 1999

**Authors:** Masahiro Kawaida, Hiroyuki Fukuda, Naoyuki Kohno

**Affiliations:** 1 Department of Otolaryngology Tokyo Metropolitan Ohtsuka Hospital 2-8-1, Minamiohtsuka Toshima-ku Tokyo 170-0005 Japan; 2 Department of Otolaryngology Keio University School of Medicine 35 Shinanomachi Shinjuku-ku Tokyo 160-0016 Japan; 3 Department of Otolaryngology National Defense Medical College 3-2, Namiki Saitama Tokorozawa 359-0042 Japan

## Abstract

A case of squamous cell carcinoma with sarcomatous stroma of the mesopharynx is presented. The patient was a 62-year-old man who complained of a foreign body sensation. Endoscopic examination revealed a large pedunculated mass arising from the posterior wall of the mesopharynx. The lesion was surgically resected, using a cutting snare by the endo-oral approach, and was completely removed. A diagnosis of squamous cell carcinoma with sarcomatous stroma was made histopathologically. The clinicopathological features of this case are described and compared with those of previously reported cases.


*Diagnostic and Therapeutic Endoscopy*, Vol. 5, pp. 125–130
Reprints available directly from the publisher
Photocopying permitted by license only

© 1999 OPA (Overseas Publishers Association) N.V.
Published by license under
the Harwood Academic Publishers imprint,
part of The Gordon and Breach Publishing Group.
Printed in Singapore.

### Squamous Cell Carcinoma with Sarcomatous Stroma of the Mesopharynx

MASAHIRO KAWAIDA[a,*], HIROYUKI FUKUDA[b] and NAOYUKI KOHNO[c]

[a]*Department of Otolaryngology, Tokyo Metropolitan Ohtsuka Hospital, 2-8-1, Minamiohtsuka, Toshima-ku, Tokyo 170-0005, Japan;* [b]*Department of Otolaryngology, Keio University School of Medicine, 35 Shinanomachi, Shinjuku-ku, Tokyo 160-0016, Japan;* [c]*Department of Otolaryngology, National Defense Medical College, 3-2, Namiki, Tokorozawa, Saitama 359-0042, Japan*

*(Received 8 November 1997; Revised 10 February 1998; In final form 30 May 1998)*

**A case of squamous cell carcinoma with sarcomatous stroma of the mesopharynx is presented. The patient was a 62-year-old man who complained of a foreign body sensation. Endoscopic examination revealed a large pedunculated mass arising from the posterior wall of the mesopharynx. The lesion was surgically resected, using a cutting snare by the endo-oral approach, and was completely removed. A diagnosis of squamous cell carcinoma with sarcomatous stroma was made histopathologically. The clinicopathological features of this case are described and compared with those of previously reported cases.**

*Keywords:* Squamous cell carcinoma, Sarcomatous stroma, Spindle cell carcinoma, Mesopharynx, Pedunculated tumor

### INTRODUCTION

Squamous cell carcinoma with sarcomatous stroma of the upper aerodigestive tract is a very rare tumorous lesion. This neoplasm of the head and neck is also known as spindle cell carcinoma, spindle cell squamous cell carcinoma, pseudosarcoma, pleomorphic carcinoma, carcinosarcoma, sarcomatoid squamous cell carcinoma, collision tumor or Lane tumor. A patient with this neoplasm of the mesopharynx was recently treated at our clinic. We report herein our findings in this patient who presented as a large pedunculated mass on the posterior wall of the mesopharynx. The clinicopathological features of this case are described and compared with those of previously reported cases.

### CASE REPORT

### Clinical Course

A 62-year-old man with no history of smoking or alcohol abuse presented with a one-month history

* Corresponding author. Tel.: +81-3-3941-3211. Fax: +81-3-3941-9557.

of a foreign body sensation in his throat. His past medical history was also unremarkable. Physical examination was remarkable for the presence of a large dumbbell-shaped mass filling the inferior mesopharyngeal area. The mass was visible through the oral cavity. Rhinolaryngeal flexible fiberscopy also revealed a large pedunculated mass arising from the posterior wall of the inferior portion of the mesopharynx (Fig. 1(a)). The glottis was obscured from view due to the size of the lesion. Then, the larynx and hypopharynx were endoscopically examined through the tip of a fiberscope around the tumorous lesion but no markedly abnormal findings were noted. There were no palpable lymph nodes in the neck.

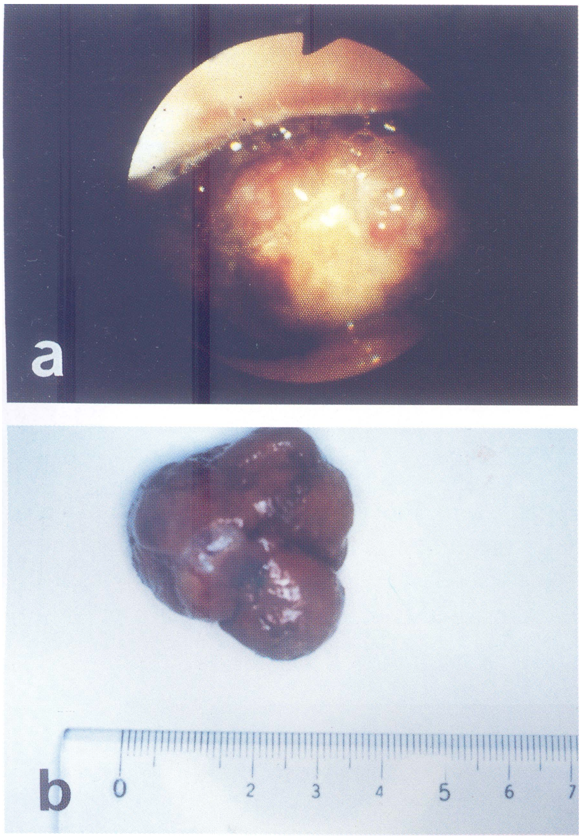

FIGURE 1 Flexible laryngofiberscopic findings at initial presentation and resected specimen. (a) A large pedunculated spherical mass arising from the posterior wall of the mesopharynx is visible. (b) Resected specimen showing a brown-colored, elastic hard and dumbbell-shaped tumor.

Computed tomography (CT) with iodide contrast medium and T2 weighted magnetic resonance imaging (MRI) with gadolinium demonstrated an approximately $3.5 \times 3.0$ cm enhanced mass with attachment to the right side of the mesopharynx (Fig. 2(a) and (b)). Although punch biopsy was performed through the oral cavity, histopathological examination revealed only necrotic tissue.

On admission, surgical resection was performed through oral approach under inhalation anesthesia by fiber-optic guided endotracheal intubation. A cutting tonsil snare was used to resect the stalk at its base because the large mass was attached to the right side of the posterior wall of the inferior mesopharyngeal area by a thin stalk, as observed intraoperatively. The tumorous mass was diagnosed histopathologically as a squamous cell carcinoma with sarcomatous stroma. Since no mucosal involvement in the resected base of the tumor was noted histopathologically and all

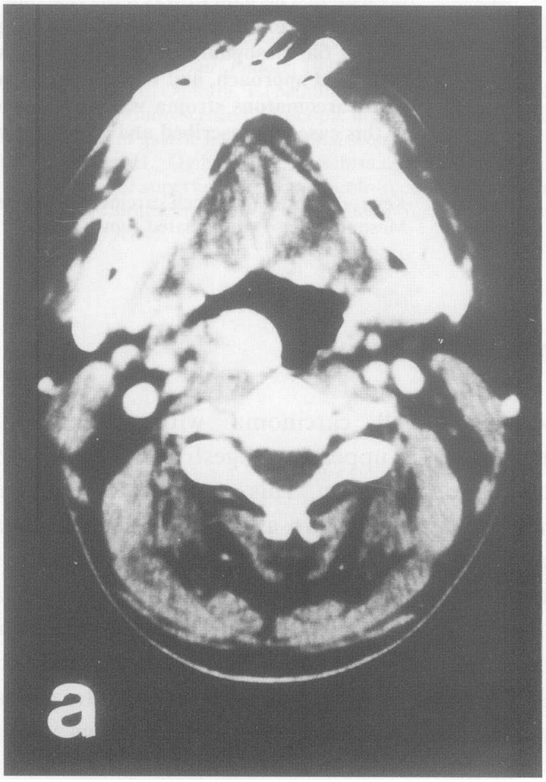

FIGURE 2(a)

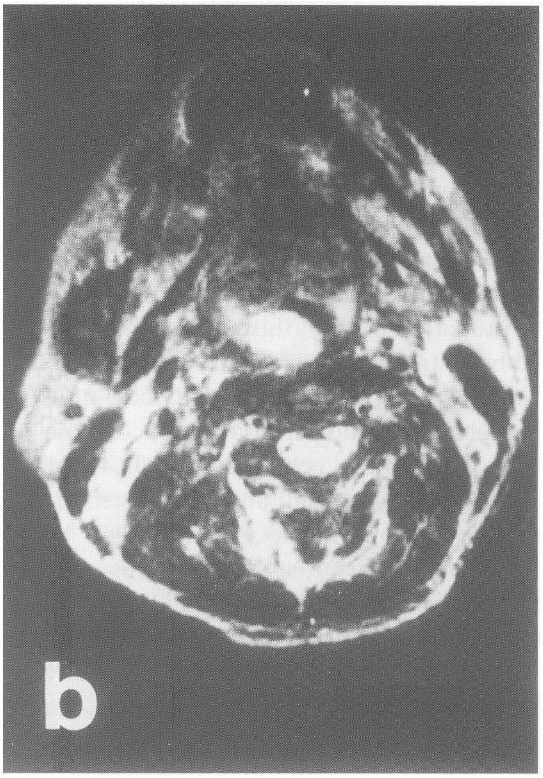

FIGURE 2(b)

FIGURE 2 CT scan and MRI. (a) Computed tomogram with contrast medium demonstrates enhanced mass arising in the right side of the mesopharynx. (b) T2 weighted magnetic resonance image in axial projection shows the lesion with high signal in the right side of the mesopharynx.

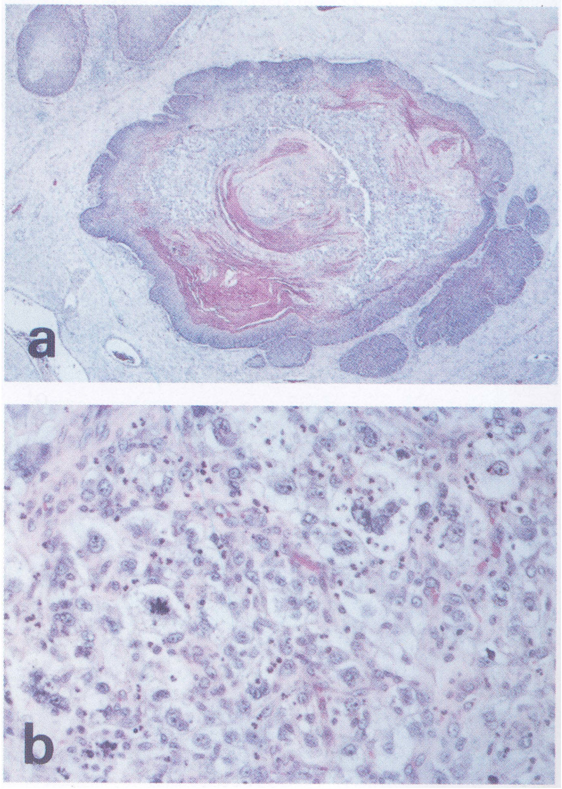

FIGURE 3 Histopathological findings of the lesion stained with hematoxylin and eosin. (a) A low-power microscopic view (× 40). (b) A high-power microscopic view of the stroma (× 100).

further staging examinations revealed no findings suggestive of metastasis outside the pharynx, the patient was discharged and followed up on an outpatient basis. No recurrence has been observed during the 37-month follow-up period.

### Histopathological Findings

The resected specimen was approximately 3.5 × 3.0 × 2.0 cm in size, brown-colored, elastic hard and dumbbell-shaped (Fig. 1(b)). Microscopic examination indicated that the specimen was lined with stratified squamous epithelium with partly ulcerative necrosis. The bulk of the lesion that stained with hematoxylin and eosin (HE) revealed foci of squamous cell carcinoma surrounded by stromal proliferation with atypical and pleomorphic cells (Fig. 3(a)). In the stroma, there was a proliferative mixture of multinucleated giant cells, round atypical cells and bizarre cells with pleomorphic nuclei and foamy cytoplasms (Fig. 3(b)). Many mitoses were also seen in the stromal cells. Immunohistochemical examinations with monoclonal antibodies to keratin, vimentin, desmin, smooth muscle actin (SMA) and S-100 protein were also performed by the avidin–biotin complex immunoperoxidase method. Keratin was positive only in parts of the squamous cell carcinoma. Vimentin and SMA, on the other hand, stained positive only in the cellular component of the stroma (Fig. 4(a) and (b)). Desmin and S-100 protein were completely negative in all

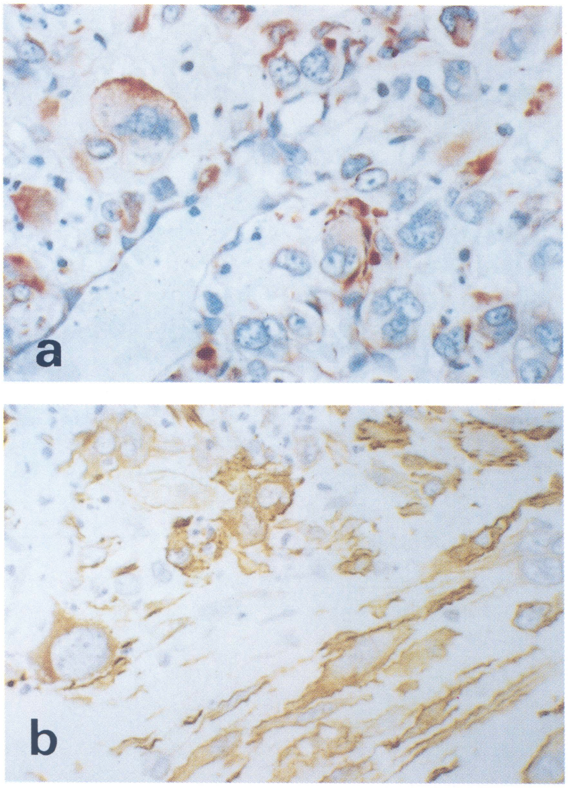

FIGURE 4 Immunocytochemical findings of the lesion. (a) Positive reaction for vimentin in the stroma (× 200). (b) Positive reaction for smooth muscle actin (SMA) in the stroma (× 200).

parts of the lesion. Based on the above findings, a histopathological diagnosis of squamous cell carcinoma with sarcomatous stroma was made in this case.

### DISCUSSION

As regards squamous cell carcinoma with sarcomatous stroma, various descriptive diagnoses have been used to term this neoplasm, such as spindle cell carcinoma, spindle cell squamous cell carcinoma, pseudosarcoma, pleomorphic carcinoma, carcinosarcoma, sarcomatoid squamous cell carcinoma, collision tumor, Lane tumor, squamous cell carcinoma with sarcoma like stroma, polypoid carcinoma, etc. [1]. This lesion is a peculiar, biphasic neoplasm that occurs mainly in the upper aerodigestive tract. Many cases with this neoplasm clinically present a pedunculated mass attached to the mucous membrane by a stalk and the tumor surface is frequently ulcerated [1,2]. It is now generally recognized that this neoplasm is a variant of squamous cell carcinoma in which a pseudosarcomatous component dominates the microscopic appearance of the neoplasm [1].

Histopathologically, this neoplasm is characterized by a stroma with pleomorphic spindle-shaped cells and giant cells making a sarcomatous pattern, mostly, but not invariably concomitant with areas of squamous cell carcinoma [3,4]. Mitoses are usually easy to find, and some osteoid, chondroid or osseous metaplasia may also be seen [1,5]. Occasionally, foci of squamous cell carcinoma are seen within the central area of stromal proliferation with pleomorphic cells [1,6].

Histopathological findings were essentially similar to those of our case described above. Keratin, a marker of epithelial properties, was positive in the squamous cell carcinoma parts. Vimentin and SMA, markers of mesenchymal properties, were positive in the stroma surrounding the carcinoma parts. The presence of SMA, in particular, indicated smooth muscle properties. Accordingly, well differentiated squamous cell carcinoma with leiomyosarcomatous stroma was seen in the histopathological examinations utilizing immunohistochemical stains. Because the stromal cells were chiefly composed of round cells and giant cells without spindle cells, it did not seem appropriate to term this lesion a "spindle cell carcinoma" in the present case. Therefore, the histopathological diagnosis of "squamous cell carcinoma with sarcomatous stroma" was chosen instead. Nevertheless, the other histopathological and clinical features of this case with a pedunculated mass with an ulcerative tumor surface appeared to be consistent with those of many of the spindle cell carcinomas arising in the upper aerodigestive tract which have been reported to date [1,4,6–8].

The observation that a squamous cell carcinoma of epithelial origin and a sarcoma of mesenchymal origin appear to coexist in the same tumor is the distinct feature of this malignant neoplasm. There have been a variety of arguments concerning the origin of the sarcomatous stroma. A benign tissue reaction in the stroma of the tumor was suggested in the past [9,10]. However, the suggestion that it represented mesenchymal metaplasia of squamous cell carcinoma has recently become more convincing [11–13].

In clinical features of this neoplasm, 90% of patients are males between the fifth and ninth decades with a median age of 68 years [6]. Upper aerodigestive irritants, such as smoking and alcohol, are the predisposing risk factors [6,8]. As far as we were able to determine in our search of the relevant literature in English, 363 cases of this neoplasm of the head and neck, including our case, have so far been reported [2–4,7–37]. The most common site was the larynx ($n = 195$), consisting of the supraglottis ($n = 31$), glottis ($n = 113$), subglottis ($n = 5$), transglottis ($n = 7$), and unspecified sites ($n = 39$). This was followed by the oral cavity ($n = 93$), consisting of the lip ($n = 33$), tongue ($n = 25$), gingiva ($n = 15$), floor of the mouth ($n = 8$), buccal mucosa ($n = 6$), hard palate ($n = 1$), retromolar area ($n = 4$), and unspecified site ($n = 1$), and then the pharynx ($n = 43$), consisting of the epipharynx ($n = 2$), mesopharynx ($n = 22$), hypopharynx ($n = 18$), and unspecified site ($n = 1$). Other sites were the nasal cavity ($n = 11$), paranasal sinuses ($n = 8$), submandibular gland ($n = 3$), parotid gland ($n = 1$), and unspecified sites of the head and neck ($n = 9$). Squamous cell carcinoma with sarcomatous stroma of the mesopharynx accounted for approximately 6.1% of the head and neck. Accordingly, the mesopharynx appears to be a comparatively unusual site of occurrence of this neoplasm.

The basic treatment of this neoplasm is surgical resection, and if cervical lymph node metastasis exists, neck dissection is also necessary [1]. Hyams *et al.* reported that follow-up of 20 cases treated surgically revealed a 60% 5-year survival [6]. On the other hand, radiotherapy alone is not recommended [6]. It is reported that metastases to lymph nodes are frequent and distant metastases have also occurred [25]. However, it is generally believed that patients with exophytic and pedunculated tumors have a better prognosis than those with ulcerative-infiltrating tumors [1]. Depth of invasion is also claimed to be an important prognostic factor [2]. In our case, the tumor presented in the form of a large mass which was attached to the right side of the posterior wall of the inferior mesopharynx by a thin stalk. However, it could be snared and resected at the base of the stalk with a cutting tonsil snare through the oral cavity because the stalk was very thin. Since no mucosal involvement of the tumor was seen histopathologically, the tumor invasion of the attached mesopharynx was considered to be very slight and superficial. Moreover, because there was no evidence of metastasis, including cervical lymph nodes, the patient was followed on an outpatient basis without subsequent radiotherapy. The follow-up period has been 36 months, and the patient's course has been favorable with no signs of recurrence or metastasis. While the above factors suggest that our patient's prognosis is good, long-term follow-up is essential.

### *References*

[1] Ferlito, A. ed., Atypical forms of squamous cell carcinoma. In: *Neoplasms of the Larynx.* New York: Churchill Livingstone, 1993: pp. 135–167.

[2] Leventon, G.S. and Evans, H.C. Sarcomatous squamous cell carcinoma of the mucous membrane of the head and neck: A clinicopathologic study of 20 cases. *Cancer* 1981; **48**: 994–1003.

[3] Brodsky, G. Carcino(pseudo)sarcoma of the larynx: The controversy continues. *Otolaryngol. Clin. North Am.* 1984; **17**: 185–197.

[4] Stone, D.M., DiMauro, J. and Clemis, J.D. Spindle-cell carcinoma of the larynx. *Ear Nose Throat J.* 1987; **66**: 56–60.

[5] Batsakis, J.G., Rice, D.H. and Howard, D.R. The pathology of head and neck tumors: Spindle cell lesions (sarcomatoid carcinomas, nodular fasciitis, and fibrosarcoma) of the aerodigestive tracts, part 14. *Head Neck Surg.* 1982; **4**: 499–513.

[6] Hyams, V.J. and Heffner, D.K. Laryngeal pathology. In: Tucker, H.M., ed., *The Larynx,* second edition. New York: Thieme Medical Publishers, 1992; pp. 35–80.

[7] Nofal, F. Spindle cell carcinoma of the nasopharynx. *J. Laryngol. Otol.* 1983; **97**: 1057–1063.

[8] Rabkin, D., Singh, J.K., Hussain, A. *et al.* Spindle cell carcinoma of the pyriform sinus: A case report. *Ear Nose Throat J.* 1995; **74**: 574–577.
[9] Lane, N. Pseudosarcoma (polypoid sarcoma-like masses) associated with squamous-cell carcinoma of the mouth, fauces, and larynx: Report of ten cases. *Cancer* 1957; **10**: 19–41.
[10] Leifer, C., Miller, A.S., Putong, P.B. *et al.* Spindle-cell carcinoma of the oral mucosa: A light and electron microscopic study of apparent sarcomatous metastasis to cervical lymph nodes. *Cancer* 1974; **34**: 597–605.
[11] Hyams, V.J. Spindle cell carcinoma of the larynx. *Can. J. Otolaryngol.* 1975; **4**: 307–313.
[12] Zabro, R.J., Crissman, J.D., Venkat, H. *et al.* Spindle-cell carcinoma of the upper aerodigestive tract mucosa: An immunohistologic and ultrastructural study of 18 biphasic tumors and comparison with seven monophasic spindle-cell tumors. *Am. J. Surg. Pathol.* 1986; **10**: 741–753.
[13] Slootweg, P.J., Roboll, P.J.M., Müller, H. *et al.* Spindle-cell carcinoma of the oral cavity and larynx: Immunohistochemical aspects. *J. Cranio-Maxillofac Surg.* 1989; **17**: 234–236.
[14] Appelman, H.D. and Oberman, H.A. Squamous cell carcinoma of the larynx with sacoma-like stroma: A clinicopathologic assessment of spindle cell carcinoma and "pseudosarcoma". *Am. J. Clin. Pathol.* 1965; **44**: 135–145.
[15] Lichtiger, B., Mackay, B. and Tessmer, C.F. Spindle-cell variant of squamous carcinoma: A light and electron microscopic study of 13 cases. *Cancer* 1970; **26**: 1311–1320.
[16] Goellner, J.R., Devine, K.D. and Weilend, L.H. Pseudosarcoma of the larynx. *Am. J. Clin. Pathol.* 1973; **59**: 312–326.
[17] Randall, G., Alonso, W.A. and Ogura, J.H. Spindle, cell carcinoma (pseudosarcoma) of the larynx. *Arch. Otolaryngol.* 1975; **101**: 63–66.
[18] Friedel, W., Chambers, R.G. and Atkins, J.P.Jr., Pseudosarcoma of the pharynx and larynx. *Arch. Otolaryngol.* 1976; **102**: 286–290.
[19] Battifora, H. Spindle cell carcinoma: Ultrastructural evidence of squamous origin and collagen production by the tumor cells. *Cancer* 1976; **37**: 2275–2282.
[20] Howell, J.H., Hyams, V.J. and Sprinkle, P.M. Spindle cell carcinoma of the nose and paranasal sinuses. *Surg. Forum* 1978; **29**: 565–568.
[21] Sismanis, A., Polisar, I.A., Santos, V. *et al.* Spindle-cell carcinoma of the laryngopharynx. *Ear Nose Throat J.* 1978; **57**: 23–537.
[22] Ellis, G.L. and Corio, R.L. Spindle cell carcinoma of the oral cavity: A clinicopathologic assessment of fifty-nine cases. *Oral Surg.* 1980; **50**: 523–534.
[23] Alguacil-Garcia, A., Alonso, A. and Pettigrew, N.M. Sarcomatoid carcinoma (so-called pseudosarcoma) of the larynx simulating malignant giant cell tumor of soft parts: A case report. *Am. J. Clin. Pathol.* 1984; **82**: 340–343.
[24] Leuszler, R.W., Shapshay, S.M. and Strong, M.S. Conservation surgery for laryngeal pseudosarcoma. *Otolaryngol Head Neck Surg.* 1984; **92**: 480–484.
[25] Recher, G. Spindle cell squamous carcinoma of the larynx: clinico-pathological study of seven cases. *J. Laryngol. Otol.* 1985; **99**: 871–879.
[26] Ophir, D., Marshak, G. and Czernobilsky, B. Distinctive immunohistochemical labeling of epithelial and mesenchymal elements in laryngeal pseudosarcoma. *Laryngoscope* 1987; **97**: 490–494.
[27] Ellis, G.L., Langloss, J.M., Heffner, D.K. *et al.* Spindle-cell carcinoma of the aerodigestive tract: An immunohistochemical analysis of 21 cases. *Am. J. Surg. Pathol.* 1987; **11**: 335–342.
[28] Meijer, J.W.R., Ramaekers, F.C.S., Manni, J.J. *et al.* Intermediate filament proteins in spindle cell carcinoma of the larynx and tongue. *Acta Otolaryngol.* (*Stockholm*) 1988; **106**: 306–313.
[29] Hellquist, H. and Olofsson, J. Spindle cell carcinoma of the larynx. *APMIS* 1989; **97**: 1103–1113.
[30] Toda, S., Yonemitsu, N., Miyabara, S. *et al.* Polypoid squamous cell carcinoma of the larynx: An immunohistochemical study for ras p21 and cytokeratin. *Path. Res. Pract.* 1989; **185**: 860–866.
[31] Takata, T., Ito, H., Ogawa, I. *et al.* Spindle cell squamous carcinoma of the oral region: An immunohistochemical and ultrastructural study on the histogenesis and differential diagnosis with a clinicopathological analysis of six cases. *Virchows Archiv. A Pathol. Anat.* 1991; **419**: 177–182.
[32] Park, Y.W. and Clarke, R.E. Spindle cell carcinoma of the larynx with simultaneous carcinoma of the thyroid gland. *Am. J. Otolaryngol.* 1993; **14**: 350–353.
[33] Larsen, E.T., Duggan, M.A. and Inoue, M. Absence of human papilloma virus DNA in oropharyngeal spindle cell squamous carcinoma. *Am. J. Clin. Pathol.* 1994; **101**: 514–518.
[34] Cassidy, M., Maher, M., Keogh, P. *et al.* Pseudosarcoma of the larynx: The value of ploidy analysis. *J. Laryngol. Otol.* 1994; **108**: 525–528.
[35] Ishibashi, T. and Hojo, H. Spindle cell carcinoma of the parotid gland. *J. Laryngol. Otol.* 1995; **109**: 683–686.
[36] Grossl, N., Tadros, T.S. and Naib, Z.M. Sarcomatoid carcinoma of the larynx with neck and distant subcutaneous metastases: A case report with fine needle aspiration cytology. *Acta Cytol.* 1996; **40**: 756–760.
[37] Lewis, J.E., Olsen, K.D. and Sebo, T.J. Spindle cell carcinoma of the larynx: Review of 26 cases including DNA content and immunohistochemistry. *Hum. Pathol.* 1997; **28**: 664–673.

